# Graves disease is more prevalent than Hashimoto disease in children and adolescents with type 1 diabetes

**DOI:** 10.3389/fendo.2022.1083690

**Published:** 2023-01-10

**Authors:** Lu-Ting Wang, Chi-Yu Huang, Chao-Hsu Lin, Bi-Wen Cheng, Fu-Sung Lo, Wei-Hsin Ting, Yann-Jinn Lee

**Affiliations:** ^1^ Department of Pediatric Endocrinology, MacKay Children’s Hospital, Taipei, Taiwan; ^2^ Department of Pediatric Endocrinology, Hsinchu MacKay Memorial Hospital, Hsinchu, Taiwan; ^3^ Department of Biological Science and Technology, National Yang Ming Chiao Tung University, Hsinchu, Taiwan; ^4^ Department of Pediatrics, Chang Gung Memorial Hospital, Taoyuan, Taiwan; ^5^ College of Medicine, Chang Gung University, Taoyuan, Taiwan; ^6^ Department of Medicine, MacKay Medical College, New Taipei City, Taiwan; ^7^ Department of Medical Research, Tamsui MacKay Memorial Hospital, New Taipei City, Taiwan; ^8^ Institute of Biomedical Sciences, MacKay Medical College, New Taipei City, Taiwan; ^9^ Department of Pediatrics, School of Medicine, College of Medicine, Taipei Medical University, Taipei, Taiwan

**Keywords:** autoimmune thyroid disease, Graves disease, Hashimoto disease, child, type 1 diabetes

## Abstract

**Introduction:**

Autoimmune thyroid disease (AITD) is the most common associated autoimmune disorder in type 1 diabetes (T1D). Early detection of AITD is crucial to optimize glycemic control, growth, and intellectual development. In this prospective cohort study, we sought to characterize the prevalence, incident ages and risk factors of AITD in children and adolescents with T1D.

**Materials and methods:**

Patients with T1D diagnosed at ≤ 18 years at MacKay Children’s Hospital, Taipei, from 1990 to 2019 underwent annual screening for AITD. Institutional Review Board-approved data on age, sex, and disease profile are collected. Statistical analysis was performed by using independent sample t test for continuous variables, chi-squared test for categorical variables, and Kaplan-Meier estimates of cumulative incidence of AITD were calculated. A *p* value of <0.05 was considered statistically significant.

**Results:**

We prospectively followed up 808 patients with T1D, 761 patients were included in the study. Of these patients, 197 (25.9%) of them had thyroid autoimmunity, meaning positivity of thyroid autoantibodies. Females had a higher prevalence of thyroid autoimmunity than males (59.9%, p = 0.012). Altogether, 5.5% patients developed AITD (4.1% had Graves disease; 1.4% had Hashimoto disease), at a mean age of 17.8 ± 8.5 years. The cumulative incidence of AITD at 30 years of disease duration was 0.29 in the total group and was significantly higher in females (0.39, n = 397) than in males (0.15, n = 364, *p*<0.001).

**Discussion:**

In Taiwan, the prevalence of AITD in pediatric population with T1D increases with age, a longer disease duration and female sex. For early detection of autoimmune thyroid disease in Taiwanese children and adolescents with T1D, an annual AITD screening program should be implemented.

## Introduction

Type 1 diabetes (T1D) is among the most common endocrine disorders in children and adolescents worldwide (1). The estimated incidence of T1D among those 19 years or younger vary in global population, ranging from 35.7 per 100,000 person years in the Nordic countries to 1.93 per 100,000 person years in China (2, 3). The variation in the incidence of T1D among major ethnic populations demonstrate a different degree of genetic and environmental susceptibility to the disease (4). Over the past decades, secular trends toward increasing incidence of T1D have been observed across the globe (5). In Taiwan, the incidence rate for the population younger than 18 years have risen from 4.84 to 5.17 per 100,000/year from year 2005 to 2014 (6).

Since the introduction of National Health Insurance in 1995, better public healthcare has improved life expectancy in T1D population (7), but the management of diabetic complications is still challenging. Additional autoimmune diseases add complexity to the management of T1D and impair the quality of life in these patients (8). A recent Finnish nationwide study reported every fifth individual with T1D suffers from an additional autoimmune disease (9). In a pediatric cohort, greater occurrence of autoimmune disease has been observed in late childhood (10). Autoimmune thyroid disease (AITD) may develop in 15 to 30% of patients with T1D during follow-up and is the most common autoimmune disorder associated with T1D (11, 12). Data from 28,671 T1D patients in Germany/Austria between 1990 and 2008 revealed 19.6% having thyroid autoimmunity (13). The prevalence of thyroid autoantibodies in patients with T1D was also significantly higher than in the general population (14, 15). The thyroid autoimmunity in T1D has important clinical implications in the patient care and understanding of T1D-associated autoimmunity. Hyperthyroidism enhances hepatic gluconeogenesis, increases insulin resistance, and may precipitate hyperglycemia and ketoacidosis. In hypothyroidism, peripheral glucose assimilation is delayed, liver andmuscle gluconeogenesis and glycogenolysis, as well as insulin clearance rate are reduced, leading to hypoglycemic events (16, 17).

Previous studies reported the incidence rate of AITD in T1D in Taiwanese youths was 1032.3 per 100,000 person years, but research on the demographic characteristic of AITD in these patients is lacking (18). Despite extensive studies on the T1D-associated autoimmune diseases in young age-groups, there is limited understanding about the lifetime risks of AITD in pediatric patients with T1D.

In this prospective study, we examined a large pediatric cohort in East Asia, seeking to answer three questions: 1) what the prevalence of AITD in children and adolescents with T1D is, 2) when it develops in relation to T1D onset, and 3) what factors are associated with the development of AITD.

## Materials and methods

We prospectively followed up 808 patients with T1D diagnosed between January 1 1990 to December 31 2019 at age ≤18 years in MacKay Children‘s Hospital, a tertiary referring center for T1D in Taiwan, based on clinical manifestations and laboratory evidence (11, 16). T1D was defined by 1) a fasting plasma glucose of ≥ 7 mmol/l (126 mg/dl), or 2) an HbA1c of ≥6.5%, 3) a two-hour plasma glucose of ≥11.1 mmol/l (200 mg/dl) during an oral glucose tolerance test (OGTT) or 4) a random glucose of ≥11.1 mmol/l (200 mg/dl) with classic symptoms of hyperglycemia; with the presence of one or more of the following autoimmune markers: islet cell autoantibodies, autoantibodies to glutamic acid decarboxylase (GADA), insulinoma-associated protein 2 (IA2A), zinc transporter 8 (ZnT8A) or insulin (19, 20). Patients without evidence of β-cell autoimmunity were diagnosed based on serum C-peptide of <0.7 mmol/l (2.1 ng/ml) at random or <1.1 mmol/l (3.3 ng/ml) at 6 minutes post glucagon stimulation test (21).

### Diagnosis of AITD

GD was diagnosed on the basis of clinical and laboratory evidence (thyrotoxicosis, diffuse goiter, with or without ophthalmopathy, elevated free T4/total T4, suppressed TSH levels, and positive TSH receptor antibody (TSHRAb)) (22). HD was diagnosed by the presence of goiter, hypothyroidism, and elevated thyroid peroxidase antibody (TPOAb), or thyroglobulin antibody (TGAb) (23). For the group with positivity of thyroid autoantibodies, the date of first antibody detection was recorded.

Patients were grouped into T1D without thyroid autoantibodies, T1D with thyroid autoimmunity alone, meaning positivity of thyroid autoantibodies detected on at least two occasions in the study period but still euthyroid needing no antithyroid therapy nor thyroxine replacement, and T1D with AITD, namely GD or HD. The Institutional Review Board approved the study. Written informed consent was obtained from all participants and/or guardians with participants’ assent.

### Autoantibody assay

GADA, IA2A, and IAA were measured by radioimmunoassay using 125I labeled human GAD-65, human recombinant IA2, and human insulin (125I-Tyr-A14-insulin), respectively (CIS Bio International, France). The cut-off level for positivity was set at the 99.5th percentile of control populations. The positivity was >1 U/mL for GADA, >1 U/mL for IA2A, and >5.5% for IAA. The intra-assay coefficient of variation (CV) was 3.6% for GADA, 2.6% for IA2A, and 2.4% for IAA. The inter-assay CV was 6.9% for GADA, 4.3% for IA2A, and 3.1% for IAA (24). ZnT8A was assayed with ElisaRSRTM ZnT8 Ab kit (RSR Limited, Cardiff, UK). The positivity was ≥15 u/ml. The intra-assay CV was 5.3% and inter-assay CV 8.5%. Free T4 was measured with radioimmunoassay (Beckman Coulter, Prague, Czech Republic; reference range, 0.89-1.79 ng/dl); TSH with radioimmunometric assay (Cisbio, Codolet, France; reference range, 0.25-4.00 mIU/ml); and TSHRAb with radioreceptor assay (RRA) kit (Cisbio, Cardiff, United Kingdom; reference range, <15%). TGAb and TPOAb were measured with a sandwich chemiluminescence immunoassay (Diasorin, Saluggia, Italy; reference range, 5 - 100 IU/mL for TGAb and 1-16 IU/mL for TPOAb)

### Statistical analysis

The prevalence of AITD in T1D was calculated by measuring the number of patients developed AITD between 1990 to 2019 divided by the total number of patients enrolled. Differences between groups were assessed using an independent sample t test for continuous variables or χ^2^ test for categorical variables. As a measure of the probability to develop autoimmune thyroid disease after T1D diagnosis, cumulative incidence was used and calculated using the Kaplan-Meier analysis. All analyses were performed using SAS 9.4. A *p* value of < 0.05 was considered statistically significant.

## Results

At the end of study, 47 patients were lost to follow-up and excluded from the analysis. Of the remaining 761 patients, 364 (47.8%) were males and 397 (52.2%) were females, with a mean age of 8.3 ± 4.2 years at diagnosis of T1D and a mean diabetes duration of 15.5 ± 8.0 years. Thyroid autoimmunity, including AITD, was found in 25.9% (197/761) of the patients; they were older (25.4 ± 8.0 vs. 23.2 ± 8.6 years, *p* = 0.002) and had longer disease duration (17.6 ± 7.5 vs. 14.7 ± 8.0 years, *p*<0.001), with female preponderance (59.9% vs. 49.5%, *p* = 0.012) (**Table 1**).

**Table 1:** Comparison between patients with and those without thyroid autoimmunity.

	With Thyroid Autoimmunity (including AITD)	No Thyroid Autoimmunity	p* value
Number	197	564	
female, No. (%)	118 (59.9)	279 (49.5)	0.012^#^
Current Age, y	25.4 (8.0)	23.2 (8.6)	0.002
Age at T1D diagnosis, y	7.8 (4.2)	8.5 (4.3)	0.061
Disease duration, y	17.6 (7.5)	14.7 (8.0)	<0.001

Data are mean (standard deviation) unless otherwise indicated.

*two-tailed t-test unless otherwise indicated.

^#^chi-square test.

**Figure 1:**
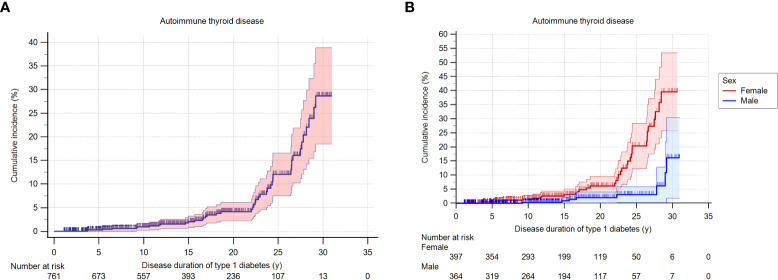
**(A)**. Cumulative incidence (probability) of AITD according to the duration of T1D in the whole group. **(B)**. Cumulative incidence (probability) of AITD according to the duration of T1D, stratified by sex of the patients.

During the study period of 1990 to 2019, 5.5% (42 of 761 patients) developed AITD, at a mean age of 17.8 ± 8.5 years (**Table 2**). Their mean diabetes duration was 19.7 ± 7.5 years. Among them, 80.9% (34/42) were females (*p*<0.001). The cumulative incidence of AITD at 30 years of disease duration was 0.29 in the whole group (n = 761) (**Figure 1A**) and was significantly higher in females (0.39, n = 397) than in males (0.15, n = 364, *p*<0.001) (**Figure 1B**), with a hazard ratio of 3.54. No association between age at diagnosis of T1D and the development of AITD was detected.

**Table 2:** Comparison between patients with AITD and those without AITD.

	AITD	With and Without Thyroid Autoimmunity	p* value
Number	42	719	
Female, No. (%)	34 (80.9)	363 (50.4)	<0.001^#^
Current Age, y	27.7 (8.3)	23.5 (8.5)	0.002
Age at T1D diagnosis, y	8.0 (4.5)	8.3 (4.2)	0.495
Age at AITD diagnosis, y	17.8 (8.5)	–	
Disease duration, y	19.7 (7.5)	15.2 (8.0)	<0.001

AITD, autoimmune thyroid disease; T1D, type 1 diabetes.

Data are mean (standard deviation) unless otherwise indicated.

*Two-tailed t-test unless otherwise indicated.

^#^Chi-square test.

Of these 42 patients, 31 (73.8%) had GD and 11 (26.2%) had HD. Compared to patients with HD, those with GD were older at diabetes diagnosis (8.8 ± 4.3 years vs. 5.6 ± 4.6 years, *p* = 0.046), and thyroid autoimmunity developed in late adolescence (18.0 ± 8.3 years vs. 9.1 ± 7.2 years, *p* = 0.003) (**Table 3**). The duration from T1D diagnosis to detection of thyroid autoimmunity was longer in patients with GD than in those with HD (9.2 ± 8.2 years vs. 3.5 ± 4.7 years, *p* = 0.038); but the duration from the detection of thyroid autoimmunity to onset of clinical disease was significantly shorter in patients with GD (0.6 ± 1.1 years vs. 6.5 ± 6.8 years, *p* = 0.016).

**Table 3:** Comparison between patients with Graves disease and those with Hashimoto disease.

	Graves Disease	Hashimoto Disease	p* value
Number	31	11	
Female, No. (%)	24 (77.4)	10 (90.9)	0.334^#^
Current Age, y	27.7 (9.1)	27.6 (6.3)	0.969
Age at T1D diagnosis, y	8.8 (4.3)	5.6 (4.6)	0.046
Age at thyroid autoimmunity, y	18.0 (8.3)	9.1 (7.2)	0.003
Age at AITD diagnosis, y	18.5(8.1)	15.7(9.6)	0.441
Diabetes duration, y	18.9 (8.4)	21.9 (3.3)	0.099
T1D onset to thyroid autoimmunity, y	9.2 (8.2)	3.5 (4.7)	0.038
Thyroid autoimmunity to AITD, y	0.6 (1.1)	6.5 (6.8)	0.016

Data are mean (standard deviation) unless otherwise indicated.

*Two-tailed t-test unless otherwise indicated.

#Chi-square test.

## Discussion

We followed up a large pediatric cohort with T1D in Taiwan from 1990 to 2019 with a disease duration spanning from 3.7 to 29.2 years. Thyroid autoimmunity was detected in 25.9% of the study cohort, with female predominance, and associated with an older age at detection of thyroid autoimmunity and a longer duration of diabetes. Within the group of patients with AITD, diabetes duration had been longer in patients with GD, in comparison to those with HD, when thyroid autoimmunity developed. Patients with GD developed symptoms of hyperthyroidismwithin a year from the detection of thyroid autoimmunity.

About one quarter of our patients with T1D had thyroid autoantibodies, with a mean follow-up period of 15 years, these were consistent with previous studies (8, 11–13, 25). Our study demonstrated an age-dependent increase in thyroid autoantibody positivity, suggesting an overall developing process of autoimmunity (15). The subjects had a longer diabetes duration compared to those without thyroid autoantibodies. These findings were compatible with results from a recent meta-analysis showing the prevalence of TPOAb increased by 3.5% with every 10-year increase in age and 7% with every 10-year diabetes duration (26). The lack of association of age at diagnosis of T1D with thyroid autoimmunity is also consistent with published data in a group of 399 Belgian children with T1D (27).

The AITD prevalence of 5.5% in our study differed from those of 17-30% reported in literature (1, 17). The heterogeneity in rates of AITD might be the variability in study design, ethnicity, assays, and definition of the disease (28). In a study of 491 children with T1D, the onset of AITD was recorded as the date of TSH abnormalities in addition to positivity of thyroid autoantibodies; while other studies defined AITD solely from presence of thyroid autoantibodies despite clinically silent disease (10, 29). We stringently defined patients with AITD as either GD or HD being having thyroid autoimmunity with clinical and laboratory evidence of hyper-/hypothyroidism might have rendered the lower prevalence of AITD.

It seems that age, female sex, and disease duration are associated in the co-existence of T1D and AITD. Although T1D did not preferentially affect males or females in our study cohort, females had a higher risk of AITD development compared to males, with a hazard ratio of 3.54. Despite gender neutrality or slight male predominance in some countries, islet autoimmunity increases the risk of AITD regardless of gender specificity (9, 30). Increased risks of insults with older age at analysis and a longer diabetes duration were observed in patients with thyroid autoimmunity as well as in patients with AITD.

In terms of additional autoimmune diseases, most Western studies report the highest rates of HD in pediatric population with T1D (9–11, 31). In this study, we found that GD occurred more frequently than HD in our population with T1D, compatible with prior studies in East Asia (32, 33). A Japanese study which consisted of 57 patients with T1D, reported diagnosis of GD in 22.8% of the patients (34). Some studies in which mainly Caucasian participants were enrolled also found that GD is more common in African and Asian/Pacific participants (10, 14).

In comparison with patients with HD in our study, patients with GD were significantly older at diagnosis of T1D and at the development of thyroid autoimmunity, particularly in late adolescence; a longer duration from T1D diagnosis to thyroid autoimmunity was also observed. Most patients progressed to symptomatic GD in less than a year, showing that TSHRAb is a strong indicator that may predict disease onset within months of antibody positivity. Although studies are few regarding GD in relation to T1D, they suggest that the progression from thyroid autoimmunity to GD takes a variable period of time (31, 35, 36). In an Italian study consisting of 1323 Caucasian pediatric patients with T1D, GD developed in 0.53% of patients a few years after T1D diagnosis at a mean age of 15 years (35). Other studies found GD was diagnosed at different stages of diabetes (37, 38). These observations demonstrate clinical characteristics of AITD in T1D differ across ethnic groups and regions.

Our research is the first prospective study in East Asia following up a large cohort of pediatric patients with T1D for almost 30 years and addresses the clinical characteristics of AITD in T1D. Our findings are significant and contribute to the epidemiology of AITD in pediatric T1D population in East Asia. It is the first study reporting the prevalence of GD in pediatric population with T1D and positivity of TSHRAb may predict the onset of clinical disease in less than a year. It highlights the importance of screening for GD in late adolescence.

This study is limited as it was performed at a single center and 47 patients were lost to follow-up during the study period. However, the design was prospective and enrolled a relatively large pediatric cohort. Although the prevalence of HD might be underestimated due to stringent inclusion criteria, the main purpose of our study is to elucidate overt hypothyroidism in HD; diagnosis based on having thyroid autoimmunity alone might be insufficient to diagnose the condition (39).

In this prospective cohort study, the prevalence of AITD in children and adolescents with T1D increases with age, female sex, and a longer disease duration. GD was more common than HD in Taiwanese children with T1D, and patients with GD progressed to clinical disease within one year of TSHRAb appearance. To optimize patient care, regular screening for thyroid autoantibodies may recognize AITD sooner in patients with T1D.

## Data availability statement

The raw data supporting the conclusions of this article will be made available by the authors, without undue reservation.

## Ethics statement

The studies involving human participants were reviewed and approved by MacKay Memorial Hospital, Taipei, Taiwan. Written informed consent to participate in this study was provided by the participants’ legal guardian/next of kin.

## Author contributions

L-TW conceptualized and designed the study, carried out the initial analyses, drafted the initial manuscript, and reviewed and revised the manuscript. Y-JL designed the data collection instruments, collected data, carried out the initial analyses, and critically reviewed and revised the manuscript. C-YH, C-HL, F-SL and B-WC coordinated and supervised data collection, and critically reviewed the manuscript for important intellectual content. W-HT conceptualized and designed the study, carried out the initial analyses, and reviewed and revised the manuscript. All authors approved the final manuscript as submitted and agree to be accountable for all aspects of the work. All authors contributed to the article and approved the submitted version.
